# The prevalence, risk factors and antifungal sensitivity pattern of oral candidiasis in HIV/AIDS patients in Kumba District Hospital, South West Region, Cameroon

**DOI:** 10.11604/pamj.2020.36.23.18202

**Published:** 2020-05-19

**Authors:** Ngwa Fabrice Ambe, Njunda Anna Longdoh, Patience Tebid, Tanyi Pride Bobga, Claude Ngwayu Nkfusai, Sangwe Bertrand Ngwa, Frankline Sanyuy Nsai, Samuel Nambile Cumber

**Affiliations:** 1 Department of Medical Laboratory Sciences, Faculty Health Sciences, University of Buea, Buea, Cameroon; 2 Department of Microbiology and Parasitology, Faculty of Science, University of Buea, Buea, Cameroon; 3 Cameroon Baptist Convention Health Service(CBCHS), Yaoundé, Cameroon; 4 Office of the Dean, Faculty of Health Sciences, University of the Free State, Bloemfontein, South Africa; 5 Centre for Health Systems Research & Development, University of the Free State, Bloemfontein, South Africa; 6 School of Health Systems and Public Health, Faculty of Health Sciences, University of Pretoria, Pretoria, South Africa

**Keywords:** HIV/AIDS, oral candidiasis, CD4 cell, antifungal

## Abstract

**Introduction:**

Oral candidiasis is one of the most common opportunistic infection in HIV/AIDS patient and it is caused by *Candida* species. The low absolute CD4+T-lymphocyte count has traditionally been cited as the greatest risk factor for the development of Oral Candidiasis. The aim of this study was to identify *Candida* species isolated from the oral cavity of HIV/AIDS patients, to determine their in vitro antifungal susceptibility and to investigate the possible risk factors associated with oral candidiasis.

**Methods:**

This was a hospital based cross sectional study that was carried out for a period of 3 months amongst HIV/AIDS patients in Kumba District Hospital, whether on HAART or not. Mouth swabs were collected from 378 participants using sterile cotton wool swabs and 5ml venous blood were collected for determination of CD4 cell. *Candida* species were isolated and identified. Antifungal sensitivity testing was performed using modified kirby-bauer susceptibility testing technique.

**Results:**

*Candida* species were present in 42.86% of the samples and *Candida albicans* was the most prevalent (60.2%) amongst the six *Candida* isolates identified, followed by *Candida glabrata* (16.9%), *Candida krusei* (12.3%), *Candida tropicalis* (6.4%), *Candida parapsilosis* (2.3%) and *Candida pseudotropicalis* (1.8%). Pregnancy, oral hygiene and antibiotic usage were significantly associated with oral candidiasis in HIV/AIDS patients (P<0.05). Oral candidiasis was mostly frequent in HIV/AIDS patients between 21-40 years. A CD4 cell count less than 200 cells/μl was a significant risk factor for acquiring oral candidiasis in HIV/AIDS patients (P<0.001). Nystatin was the most sensitive drug (83.6%) meanwhile ketonazole was the most resistant drug (29.2%), followed by fluconazole (24.6%) to all oral *Candida* isolates.

**Conclusion:**

Oral *Candida* colonization occurs more frequently in HIV/AIDS patients and the is a need for the government to implement regular checks for opportunistic infections in HIV/AIDS patients, including oral candidiasis in HIV/AIDS patients to monitor disease progression and prevent subsequent complications such as candidemia and diarrhea.

## Introduction

The acquired immune deficiency syndrome (AIDS) is caused by the human immunodeficiency virus (HIV) and is characterised by progressive damage to the immune system, is the most important public health problem of the 20^th^ century. HIV/AIDS continues to spread globally and remains a worldwide pandemic affecting about 36.7 million people [1]. It still continues to grow in countries of all incomes with a greater impact on the more vulnerable developing world and has emerged as a global crisis since its discovery in the summer of 1981[1, 2]. Even though the prevalence of HIV among adults is remarkably small (3.4%) [3] in Cameroon as compared with other sub-Sahara African countries like South Africa (19%) and Zambia (12.5%) [1, 3], it still remains a measure public health problem. Owing to a weakened immune system, the infected person is placed at an increased risk of a wide variety of opportunistic infections [4, 5]. Oral candidiasis (OC) is one of the most common fungal opportunistic infections in immunocompromised individuals [6]. Oral candidiasis occurs in up to 95% of human immunodeficiency virus (HIV)-infected individuals during the course of their illness [6, 7] and is a prognostic indicator for acquired immune deficiency syndrome (AIDS) [6, 7]. Worldwide, it is estimated that 9.5 million HIV patients suffer from oral candidiasis [6, 8]. *Candida species* normally colonise the gastrointestinal tract of healthy individuals and in HIV/AIDS patients. The infection is commonly acquired endogenously except in a few cases where strains can be transmitted from person to person [9].

Oral candidiasis is mainly caused by *Candida albicans,* which accounts for up to 81% of cases among HIV-infected individuals [6]. However, *non-albicans Candida species* have been implicated in colonization of the oral cavity, eventually causing infection in 20-40% of immunocompromised individuals [6]. Many factors have been suggested as risk factors for oral colonization; these include diabetes mellitus, head and neck cancer, smoking, the use of oral prostheses, age, race and poor nutritional status [10]. Others are reduced salivary flow, the use of antibiotics, immunosuppressive states (pregnancy) and even the presence of other locally available microflora [10]. In HIV-positive individuals, it has also been observed that the low CD4+ count, high HIV viral load, non-availability or non-usage of highly active antiretroviral therapy (HAART) and the type of antiretroviral (ARV) medication have been explored as possible factors that influence oral *Candida* carriage as well as with oral candidiasis in HIV patients [10, 11]. These data suggest that a decrease in oropharyngeal *Candida* carriage and oral candidiasis in HIV/AIDS patients can be achieved by initiating patients on highly active antiretroviral treatment (HAART) without the need for specific antifungal therapy [12]. Thus a prompt diagnosis of *Candida* infection in HIV patients and assessment of the immune status can have bearing on treatment of such infections and improve the general wellbeing of the PLWHA (people living with HIV/AIDS) [12]. The low absolute CD4+ T-lymphocyte count has traditionally been cited as the greatest risk factor for the development of oral candidiasis and current guidelines suggest increased risk once CD4+ T lymphocyte counts fall below 200 cells/μl [13]. Also, the increased incidence of mucosal and probably deep systemic forms of candidiasis has consequently made the use of antifungal agents the best option by clinicians so as to be able to control these pathogens [6, 14].

The widespread use of these antifungals has consequently led to an increase in antifungal resistance. Some authors have further observed a noticeable shift toward *non albicans* species with relative resistance to fluconazole and itraconazole [14]. The species spectrum and resistance of *Candida* isolates to currently available antifungal drugs is therefore a highly relevant factor because it causes important implications for morbidity and mortality [13, 14]. During the last few decades, the spectrum of infections has undergone a drastic change; organisms with minimal or no pathogenic role have emerged as potent pathogens and organisms once susceptible have become multidrug resistant as in the case of *non albicans species* [6, 13-15] and Up till now, no cure exists for HIV/AIDS. It then follows that controlling opportunistic pathogens associated to this pandemic remains one surer way of managing infected individuals who have the scourge [14-16]. The aim of this study was to identify *Candida* species isolated from the oral cavity of HIV/AIDS patients, to determine their in vitro antifungal susceptibility and to investigate the possible risk factors associated with the infection in the view to contributing to a better management of *Candida* infections in HIV/AIDS patients.

## Methods

**Study design:** this study was a hospital based cross sectional study carried out between 6^th^ April to 28^th^ June 2018. At enrolment an informed consent and assent form was obtained and each study participant was asked to complete a questionnaire which consisted of socio-demographic and personal details, history of present illness, clinical signs and symptoms, and so forth. Participant on HAART were identified as with or without protease inhibitor.

**Study area:** this study was conducted in Kumba District Hospital HIV/AIDS treatment center which has about 3860 active HIV/AIDS patient on treatment, which is located in Meme Division, South West Region Cameroon.

**Sample size:** sample size was calculated from the Lorenz formula:

$$n=\frac{Z^{2}P(1-P)}{d^{2}}$$

Where n is the sample size, Z is the confidence interval (95%) Z=1.95, P is the pre-estimated prevalence of 54.1% obtained from a study carried out by Njunda *et al.* in 2013 in Cameroon and d is the margin error (5%). The sample size calculated was 378 participants.

**Study population:** all HIV positive Patients of both sexes and all age groups whether on HAART or not were eligible for this study. Only those HIV seropositive subjects who have not received any specific antifungal therapy in the preceding three months were enrolled.

**Ethical considerations:** ethical review and clearance was soughed from the Institutional review board (IRB/FHS UB) and administrative clearance from the regional delegation of public health, the Kumba Health district and from the director of the Kumba District Hospital.

**Sample collection:** mouth swabs were collected from all participants using sterile cotton wool swabs and the swab was labeled with patient code. 5ml venous blood was collected for determination of CD4 cell.

### Laboratory diagnosis

**CD4 cell count:** the blood specimen was used for determination of CD4 cell count using flow cytometry (pima counter) and the counts was categorized according to standards of the WHO, as severe when counts <200 cells/μl; low (200-349 cells/μl);moderate(350-499 cells/μl) and high when counts ≥500 cells/μl [17].

**Microscopy:** oropharyngeal swab samples were microscopically examined on a clean grease free glass slide using the 10X and 40Xobjectives for the presence of pus cells and of small, round to oval, thin-walled, clusters of budding yeast cells and branching pseudohyphae characteristically typical of *Candida*. Smears were made and Gram stained and latter observed at 100X for fungi elements [18].

**Culture:** oropharyngeal swab samples were inoculated on Sabouraud dextrose agar (SDA) impregnated with Chloramphenicol and incubated at 35°C(±2°C) for 24hours under aerobic conditions for the observation of colonies which were characteristically white to cream coloured, smooth, glabrous yeast like in appearance. Colonies were Gram stained and sub-cultured on chrom agar *Candida* media and SDA impregnated with Chloramphenicol for identification and antibiogram respectively [18].

**Germ tube test:** *C.albicans* can be identified presumptively by a simple germ tube test. Single colony was inoculated in human serum and incubated for 2-4 hours at 37°C and observed for germ tube formation under the microscope using the 40x objectives [18].

**Chrom agar *Candida* medium:** the CHROM agar *Candida* (Paris, France) is a selective and differential chromogenic medium that is useful for the identification of various *Candida* species. This medium is based on direct detection of specific enzymatic activities by adding multiple chemical dyes that is substrates of fluorochromes to media. Due to the chromogenic substrates added in the medium, the *Candida* colonies of various species produce different colours, thus allowing the direct identification of these *Candida* species on the isolation plate [19]. Discrete colony were pick from the sabouroud dextrose agar impregnated with chloramphenicol an emulsified in distilled water in a sterile test tube and inoculated on chrom agar *Candida* medium by streaking the entire surface of the test plate and incubated for 18-24 hours, after 24 hours colour change was observed which indicate a particular type of *Candida* species (Table 1) [19].

**Table 1:** Identification of candida species using chrom agar medium

Sample no	Candida species	Colour on CHROM agar medium
1	Candida albicans	Light green
2	Candida glabrata	Purple
3	Candida krusei	Pink
4	Candida parapsilosis	Cream to pale pink
5	Candida tropicalis	Steel blue
6	Candida pseudotropicalis	White to mauve

**Antifungal sensitivitys testing:** antifungal susceptibility test to fluconazole (25μg), itraconazole (10μg), ketoconazole (10μg), nystatin (100μg), miconazole (50μg), Econazole(50μg), and amphotericin B (20μg) were performed by modified kirby-bauer susceptibility testing technique as described by Clinical and Laboratory Standards Institute guidelines (CLSI guidelines) (Table 2) [20].

**Table 2:** Zone diameter interpretation for fungi in millimeter

Drug Type	Potency	Sensitive	Intermediate	Resistance
Fluconzole	25μL	≥19	12-18	≤11
Miconazole	50μL	≥20	12-19	≤11
Econazole	50μL	≥20	12-19	≤11
Ketoconazole	10μL	≥28	21-27	≤20
Itraconazole	10μL	≥23	22-14	≤13
Amphotericin	10μL	≥15	10-14	≤10
Nystatin	100μL	≥15	10-14	No

**Statistical analysis:** data were recorded on register forms and entered in a Microsoft Excel database in a secure computer and analysis was done with SPSS version 20 and EPI info version 7. Data were statistically described in terms of frequencies and percentages. The Chi-squared test was applied to analyse associations of *Candida colonization with CD4 cell*, different HAART regimen and oral hygiene. Multivariate analysis was applied to analyze risk factors associated with *Candida* colonization. A P value less than 0.05 was considered statistically significant at 95% confidence interval.

## Results

**Demographic characteristics of HIV/AIDS patients in the Kumba District Hospital:** 378 HIV/AIDS patients aged range from 3-72 years were involved in this study. The mean age of the participants was 40.30(SD=14.19), the median age was 40. Majority (47.1%) of the participants falls within the age group 20-40 years and age group ≤20 constituted 6.9%. more than half (276) of the participants were females. Majority (50.3%) of the participants ended their education in primary school, 38.6% in secondary school and 11.1% in the university. Most of the participants (61.1%) said that they wash their mouth once daily (Table 3).

**Table 3:** The demographic characteristics of HIV/AIDS patients in Kumba District Hospital (n=378)

Variable	Frequency	Percentage (%)
**Age**	**Mean±SD (40.30±14.19)**	**Range (69)**
≤ 20	26	6.9
21-40	178	47.1
41-60	144	38.1
>60	30	7.9
**Gender**		
Female	276	73.0
Male	102	27.0
**Education**		
Primary	190	50.3
Secondary	146	38.6
University	42	11.1
**Brush mouth**		
Once	231	61.1
Twice	147	38.9

**Prevalence of oral candidiasis in HIV/AIDS patients:** out of the 378 HIV/AIDS patients screened for oral candidiasis, 162 HIV/AIDS patient were positive for oral candidiasis given a prevalence of 42.86%, with female and male having a prevalence of 30.69% and 12.17% respectively.

**Frequency of *Candida* species identified:** in the total 162 samples that yielded growth of *Candida* species, single species were grown in 154 samples, mixed species in 8 samples and a total of 171 isolates were identified. *Candida albicans* had the highest frequency among all the species with a total number of 103(60.2%) compared to *non albicans species.* The *non albicans* species and their frequencies included *Candida glabrata* 29(16.9%), *Candida krusei* 21(12.3%), *Candida tropicalis* 11(6.4%), *Candida parapsilosis* 4(2.3%) and *Candida pseudotropicalis* 3(1.8%) which is the least species (Table 4). Among the 8 samples with multiple growth, *Candida albicans* was present in all the multiple growth. *Candida albicans* and *Candida tropicalis* were present in 3 samples, *Candida albicans* and *Candida pseudotropicalis* were present in 3 samples, *Candida albicans* and *Candida tropicalis* was present in a sample and *Candida albicans* and *Candida krusei* was present in a sample (Table 4).

**Table 4:** The distribution of different candida species isolated

Candida species	Frequency	Percentage (%)
Candida albicans	103	60.2
Candida glabrata	29	16.9
candida krusei	21	12.3
Candida tropicalis	11	6.4
Candida parapsilosis	4	2.3
candida pseudotropicalis	3	1.8
**Total**	171	100

**Antifungal susceptibility pattern of oral *Candida* isolates:** antifungal susceptibility pattern of 171 isolates of *Candida* species was determined (Table 5). Nystatin (83.6%) was the most sensitive drug, followed by amphotericin (76%) and miconazole (74.3%). The most resistant antifungal drugs were ketoconazole (29.2%), followed by fluconazole (24.4%). In our finding amongst, the six *Candida* species isolates, *Candida albicans* was the most sensitive *Candida* species to all the seven antifungal tested as compared to *C. non-albicans,* of the *C. non-albicans* species isolated *Candida krusei* and *Candida tropicalis* were the most resistant species to all the antifungal tested. We also observed resistance to fluconazole in 24.6% of all oral *Candida* isolates (Table 5).

**Table 5:** Antifungal susceptibility profile of oral candida isolates

	ANTIFUNGAL TESTED							
CANDIDA SPECIES	Susceptibility	AMB n(%)	KET n(%)	NYS n(%)	ITR n(%)	FLU n(%)	ECO n(%)	MIC n(%)
**C. albicans (103)**	**Sensitive**	**90(87.4)**	**76(73.8)**	**94(91.3)**	**81(78.6)**	**76(73.8)**	**79 (76.7)**	**87(84.5)**
	**Intermediate**	4(3.9)	7(6.8)	1(0.9)	6(5.8)	8 (7.8)	6 (5.8)	4 (3.9)
	**Resistance**	9(8.7)	21(20.4)	8(7.8)	16(15.3)	19 (18.4)	18 (17.5)	22(11.7)
**C.glabrata (29)**	**Sensitive**	**20(69.0)**	**13(44.8)**	**20(69.0)**	**13(44.8)**	**16(55.2)**	**15(51.7)**	**16(55.2)**
	**Intermediate**	2(6.9)	3(10.3)	3(10.3)	6(20.7)	4(13.8)	5(17.2)	5(17.2)
	**Resistance**	7(24.1)	13(44.8)	6(20.7)	10(34.5)	9(31.0)	9(31.1)	8(27.6)
**C. krusei (21)**	**Sensitive**	**10(47.6)**	**9(42.9)**	**15(71.4)**	**12(57.1)**	**10(47.6)**	**11(52.3)**	**13(61.9)**
	**Intermediate**	5(23.8)	1(4.8)	1(4.8)	3(14.3)	3(14.3)	3(14.3)	1(4.8)
	**Resistance**	6(28.6)	11(52.3)	5(23.8)	6(28.6)	8(38.1)	7(33.4)	7(33.3)
**C. parapsilosis (4)**	**Sensitive**	**3(75)**	**3(75)**	**4(100)**	**3(75)**	**3(75)**	**3(75)**	**3(75)**
	**Intermediate**	1(25)	0(0)	0(0)	0(0)	0(0)	0(0)	0(0)
	**Resistance**	0(0)	1(25)	0(0)	1(25)	1(25)	1(25)	1(25)
**C. tropicalis (11)**	**Sensitive**	**5(45.5)**	**5(45.5)**	**8(72.7)**	**6(54.5)**	**5(45.5)**	**6(54.5)**	**7(63.6)**
	**Intermediate**	2(18.2)	1(9.1)	0(0)	1(9.1)	1(9.1)	1(9.1)	1(9.1)
	**Resistance**	4(36.4)	5(45.5)	3(27.3)	4(36.4)	5(45.5)	4(36.4)	3(27.3)
**C.pseudo tropicalis (3)**	**Sensitive**	**2(66.7)**	**0(0)**	**2(66.7)**	**1(33.3)**	**1(33.3)**	**1(33.3)**	**1(33.3)**
	**Intermediate**	0(0)	1(33.3)	0(0)	1(33.3)	2(66.7)	1(33.3)	2(66.7)
	**Resistance**	1(33.3)	2(66.7)	1(33.3)	1(33.3)	0(0)	1(33.3)	0(0)
**TOTAL (171)**	**Sensitive**	**130(76)**	**108(63.2)**	**143(83.6)**	**116(67.8)**	**111(64.9)**	**115(67.3)**	**127(74)**
	**Intermediate**	14(8.2)	13(7.6)	5(2.9)	17(9.9)	18(10.5)	16(9.4)	13(7.6)
	**Resistance**	27(15.8)	50(29.2)	23(13.5)	38(21.3)	42(24.6)	40(23.4)	31(18.1)

AMB= Amphotericin B, Ket= Ketoconazole, NYS= Nystatin, FLU= fluconazole ITR= Itraconazole, ECO=Econazole, MIC =Miconazole

**Distribution of the prevalence of oral candidiasis according to sex, age and CD4 levels:** the frequency of oral candidiasis was more among females (30.69%) than in males (12.17%) in this study (Table 6). However the association between gender and the prevalence of oral candidiasis was not statistically significant (P=0.593). The frequency of oral candidiasis was highest among individuals in the aged group 21 -40 years (Table 6). However the was no significant association between infection rate with age (P=0.755). Oral candidiasis was observe more frequently among individuals with CD4 cell less than 200cells/μl (13.23%) (Table 6) However, the association between CD4 cell and the prevalence of oral candidiasis was statistically significant (P<0.001) (Table 6).

**Table 6:** Distribution of the prevalence oral candidiasis according to gender, age and CD4 levels

Variables	Prevalence of oral candidiasis n(%)	Chi-square (X2)	P-value
**Gender**			
Male	46 (12.17%)	0.286	0.593
Female	116 (30.69%)		
Total	162 (42.86%)		
**Age/years**			
≤20	11 (2.91%)	1.919	0.755
21-40	81 (21.43%)		
41-60	59 (15.61%)		
>60	11 (2.91%)		
Total	162 (42.86%)		
**CD4 cells/μl**			
<200	50 (13.23%)	50.18	<0.001
200-349	43 (11.38%)		
350-499	30 (7.94%)		
≥500	39 (10.32%)		
Total	162 (42.86%)		

**Figure 1:**
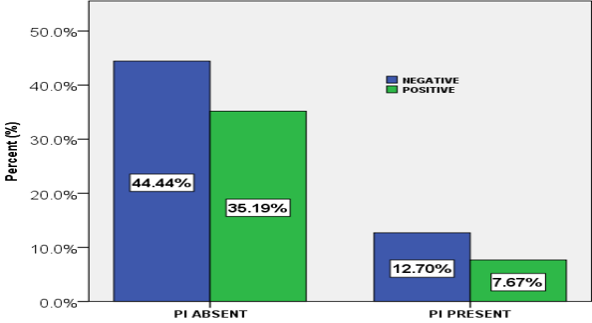
The proportion of those with oral candidiasis without protease inhibitor and those with protease inhibitor (PI)

**Association between the prevalence of oral candidiasis and protease inhibitor:** the prevalence of oral candidiasis was lower (7.67%) in patients under HAART regimen with protease inhibitor than in patients under HAART regimen without protease inhibitor (35.19%) but these difference was not statistically significant (P=0.302) (Figure 1).

**Figure 2:**
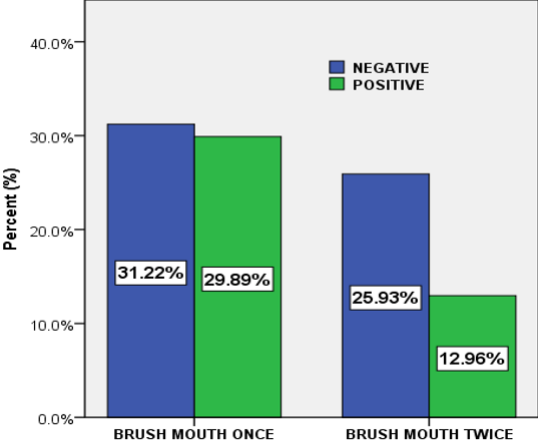
The prevalence of oral candidiasis with regard to oral hygiene

**Association between the prevalence of oral candidiasis and oral hygiene:** a low prevalence (12.96%) of oral candidiasis was noticed in patients who brush mouth twice daily and high prevalence (29.89%) of oral candidiasis in patients who brush mouth once daily. The Pearson chi square (χ^2^) test suggested that, the was a statistical significant difference between oral candidiasis and oral hygiene, (χ^2^(1)=8.909, P=0.003 (Figure 2).

**Risk factors associated to oral candidiasis in HIV/AIDS patients in Kumba District Hospital:** in the Bivariate analysis, brush mouth daily (oral hygiene) and CD4 cells/μl were significantly associated with oral candidiasis in HIV/AIDS patients. However, all variables with p-value <2 were taken to the multivariate analysis. After adjusting for confounders in the multivariate analysis, oral hygiene, pregnancy, antibiotic usage and CD4 level of the HIV/AIDS patients were significantly associated with oral candidiasis in HIV/AIDS patients (Table 7). The Odds of having oral candidiasis were significantly higher in HIV/AIDS patients who bush mouth once daily (AOR: 2.15; 95% CI: 1.32-3.50, p=0.002) compared to patients who brush mouth twice daily. Also, the Odds of having oral candidiasis were significantly higher in those that were pregnant (AOR: 2.04; 95%CI: 1.10-3.82; P=0.025) compared to those not pregnant (Table 7).

**Table 7:** Risk factors associated to oral candidiasis in HIV/AIDS patients

	Oral candidiasis			
Variable	COR (95% CI)	P-value	AOR (95%CI)	P-value
Age (years)				
**≤ 20**	1			
**21-40**	1.14 (0.50-2.62)	0.759		
**41-60**	0.95 (0.41-2.21)	0.899		
**> 60**	0.79 (0.27-2.31)	0.667		
Sex				
**Female**	1			
**Male**	1.13 (0.7-1.79)	0.591		
Level of education				
**University**	1		1	
**Secondary**	1.89 (0.91-3.92)	0.087	1.44 (0.64-3.22)	0.380
**Primary**	1.69 (0.83-3.46)	0.145	1.22 (0.55-2.69)	0.628
Brush mouth daily				
**Twice**	1		1	
**Once**	1.92 (1.25 -2.94)	0.003	2.15 (1.32-3.50)	0.002
Antibiotics usage				
**No**	1			
**Yes**	1.78(0.88-3.56)	0.108	2.2(1.03-4.74)	0.045
Pregnant				
**No**	1		1	
**Yes**	1.53 (0.86-2.72)	0.151	2.04 (1.10-3.82)	0.025
Protease Inhibitor				
**Absent**	1			
**Present**	0.76 (0.46-1.28)	0.303		
When diagnosed (Years)	A			
**≥ 5**	1			
**< 5**	0.90 (0.60-1.35)	0.613		
CD4 Cells/μl				
**< 200**	1		1	
**200-349**	0.17 (0.08-0.37)	<0.001	0.15 (0.07-0.33)	<0.001
**350-499**	0.11 (0.05-0.24)	<0.001	0.08 (0.04-0.20)	<0.001
**≥500**	0.09 (0.05-0.21)	<0.001	0.09 (0.04-0.19)	<0.001

## Discussion

Oral candidiasis (OC) is one of the most common fungal opportunistic infections in immunocompromised individuals [6]. Oral candidiasis occurs in up to 95% of human immunodeficiency virus (HIV)-infected individuals during the course of their illness [6], and is a prognostic indicator for acquired immune deficiency syndrome (AIDS) [6]. Our results showed that 42.86% oral swabs collected from the oral cavity of HIV/AIDS patients, grew *Candida* colonies. This result is in line with reports from similar studies in Brazil [21, 22], where prevalence of 41.87% and 42% were recorded respectively. This prevalence (42.86%) recorded in this study is lower than that reported by Njunda *et al.* [15] and Nweze *et al.* [14], where prevalence of 54.1% and 60% were recorded respectively, these difference could probably be due to the fact that not all participants in these studies were on HAART and the advent of HAART has allowed for the suppression of viral replication to very low levels and a partial recovery of CD4 cells in patients with HIV, which has consequently reduced opportunistic infections.[11, 21, 22].

Candida albicans was the most frequent species isolated in these study which is consistent with previous studies [6, 15, 23], and this result contrasts with the observation in some countries in the Gulf region [24]. 60.2% of Candida albicans was isolated in this study which is in line with studies reported in Brazil [22, 23]. The possibly reason why Candida albicans constitute majority of the Candida species isolated from the oral cavity(60.2%) is probably facilitated by a number of virulence factors, the most important of which are the possession of multiple adhesins for adherence to host tissues, biofilm formation and secretion of hydrolytic enzymes proteases, phospholipases and haemolysins [25]. Also C. albicans reversibly converts from unicellular yeast cells to either pseudohyphal or hyphal growth, a morphogenesis phenomenon. The growth of hyphae, plays an important function in tissue invasion and resistance to phagocytosis [25]. Non albicans species accounted for about 39.8 of Candida species from the oral cavity. This result is in conformity to 44% reported in Brazil [23] and 20-40% reported in sub Saharan Africa [6]. Among these, Candida glabrata 29(16.9%) was the most frequent, which was followed by Candida krusei 22(12.3%), Candida tropicalis 11(6.4%), Candida parapsilosis 4(2.3%) and Candida psudotropicalis 3(1.8%), is a relatively common finding with other similar studies [6, 14, 23], but there were some striking differences in the species spectrum and percentage of recovered isolates. Candida glabrata, Candida krusei and Candida tropicalis were the most frequent non albicans species isolated from the HIV/AIDS patients in these study which is consistent with a study in sub Saharan Africa [6]. This is of public health concern because some of these non albicans species are intrinsic resistant to fluconazole which is the most commonly used azoles in developing countries [26].

Treatment against candidiasis varies substantially depending on the anatomical localization of the infection, the immune status of the patient, and the isolated species [27]. In order to understand the therapeutic differences in the isolates involved in oral candidiasis study population, we tested the sensitivity of these isolates. Our finding indicated that amongst all *Candida* isolates tested, nystatin was the most sensitive antifungal drug (83.6%), followed by amphotericin (76%) and miconazole (74.3%). This finding partly agree with a similar study carried out in Mexico [28], where nystatin and amphotericin were the most sensitive antifungal tested. This finding is in contrast with a similar study carried out in Cameroon by Njunda *et al.* [15], where ketonazole was found to be the most sensitive antifungal drug (85.5%). This disparity in result is probably due to decrease sensitivity of the *Candida* species isolated to azoles. Although in general *Candida* species are susceptible to both azoles and polyenes, antifungal susceptibility varies significantly between *C. albicans* and *C. non-albicans.* In our finding amongst, the six *Candida* species isolates, *Candida albicans* was the most sensitive *Candida* species to all the seven antifungal tested as compared to *C. non-albicans.* This is probably due to the fact that *C. non-albicans* are intrinsically resistant to azoles (fluconazole) [26]. Of the *C. non-albicans* species isolated *Candida krusei* and *Candida tropicalis* were the most resistant species to all the antifungal tested.

We observed resistance to fluconazole in 24.6% of oral *Candida* isolates; this prevalence is higher than the rates obtained in different areas in Cameroon [15] and Nigeria [29] but is in consistent with the 24.0% prevalence recorded in Benin City in Nigeria [30]. The possible explanations for the difference in rates include methodological, temporal, drug usage, immune status, regional variations and intrinsic resistance of the *Candida* species [14]. In Africa, fluconazole is usually considered to be the drug of choice in both the treatment and prophylactic prevention of fungal infections in HIV-infected individuals and people with HIV/AIDS [6]. This is due to its favourable pharmacokinetic profile, low toxicity, and availability. Consequently, increase resistance to the drug poses a threat not only to the treatment of oral candidiasis HIV/AIDS patient, but also to the management of systemic fungi infections HIV/AIDS patients. The other azoles recorded resistance which was closely similar to fluconazole; this is in contrary to previous study [28]. This is probably due to cross resistance to fluconazole, since all azoles exert the same mechanism of action and fluconazole was routinely administered to HIV/AIDS patients with clinical symptoms of candidiasis without prior antifungal susceptibility testing in these center.

The carriage rate was found to be more in women (30.69%) than in men (12.17%), but these different in prevalence was not statistically significant (P=0.598). This difference in prevalence is in conformity with studies carried out in Cameroon [15] and Mexico [28]. This difference is probably because most men rarely go for routine checkups until the disease has reached symptomatic stage and as at the time of this study only a few men consented. Participants around the age group of 21-40 year accounted for the highest prevalence rate (21.43%), but the difference in prevalence rate among age groups was not observed to be statistically significant (P=0.755). This result is in line with a study carryout in Cameroon [15], but contrary to study carried out in Mexico [28] which shows that *Candida* colonization was age dependent. This highest prevalence rate between these age group (21-40 year) is probably due to the fact that, they represent majority of the study participants and which constituted the highest active sex group which may also spread oral candidiasis exogenously.

The distribution of the prevalence of oral candidiasis at different CD4 cell levels, oral candidiasis (13.23%) was more frequent in individuals with <200 cells/μl and was a common observation with other studies [16, 21, 31]. This was a statistically significant association between the prevalence of oral candidiasis and low CD4 cells (P<0.001). This difference may be related to the decrease of the immunodeficiency of the immune system. This is supported by Fidel (2006), who suggests that *Candida* specific T cells do not become defective with immunosuppression, but a threshold number of CD4 cells are required to protect the oral cavity against infection by this normal commensal [32]. The prevalence of oral candidiasis was lower (7.67%) in patients under HAART combination with protease inhibitor than in patients under HAART combination without protease inhibitor (35.19%) which is conformity with a study carried out in Nigeria, [33] but these difference in prevalence of oral candidiasis was not statistically significant (P=0.302) and may be due to the fact that protease inhibitor group of ARV drugs exerts an early immune reconstitution-independent beneficial effect against oral candidiasis, which is entirely due to the protease inhibitor capacity to inhibit Sap enzymes of *Candida* secretory aspartyl proteinase (Sap) family that belongs to the same family as HIV-proteinase [34].

The was a statistical significant difference between the prevalence of oral candidiasis and oral hygiene (χ^2^(1)=8.909, P=0.003). A low prevalence (12.96%) of oral candidiasis was noticed in patients who brush mouth twice daily and a high prevalence (29.89%) of oral candidiasis in patients who brush mouth once daily. This association between oral candidiasis and oral hygiene was not consistent in a study carried out in Nigeria [33]. This difference in prevalence is probably due to that fact that good oral hygiene; however, is an essential part of treating oral thrush. Healthy adults and children can recover fairly easily from the infection, especially if they follow a complete oral care routine of twice-daily tooth brushing and daily proper flossing. In the Bivariate analysis, brush mouth daily (oral hygiene) and CD4 cells/μl were significantly associated with oral candidiasis in HIV/AIDS patients. However, all variables with p-value <2 were taken to the multivariate analysis. After adjusting for confounders in the multivariate analysis, oral hygiene, pregnancy, antibiotic used and CD4 level of the patient were significantly associated with oral candidiasis in HIV/AIDS patients. The Odds of having oral candidiasis were significantly higher in those that were pregnant (AOR: 2.04; 95%CI: 1.10-3.82; P=0.025) compared to those not pregnant and this finding is similar to previous studies [35, 36]. This is possibly due to the fact that pregnancy lead to immune suppression, altered bacteria flora and causes hormonal in balance which are major risk factor to oral candidiasis [35, 36]. The odds of *Candida* colonization were significantly greater in patients taking antibiotics in our multivariate analysis and this is consistent with observations of other investigators [32, 35]. This is probably due to the fact that broad spectrum antibiotics (Bactrim) alter the local oral flora creating a suitable environment for *Candida* to proliferate, which was usually administered to these patients in this center.

## Conclusion

We concluded that, oral *Candida* colonization occurs more frequently in HIV/AIDS patients and are significantly more common in patients with CD4+ cell counts <200 cell/μl, thus placing them at higher risk of invasive infections caused by these pathogens. In our study a prevalence of 42.86% of oral candidiasis was obtained among HIV/AIDS patients, this prevalence among HIV/AIDS patient was not observed to be gender and age dependent in these study. A total of six *Candida* species were identified in this study, with *Candida albicans* (60.2%) been the most predominant *Candida species* identified. Polyenes (amphotericin and nystatin) recorded the highest sensitivity rate to all *Candida* species isolates. Azoles recorded almost similar sensitivity and resistance but ketonazole was the most resistant to all *Candida* isolates, followed by fluconazole and this is probably due to cross resistance to fluconazole. *Candida albicans* was the most sensitive *Candida* species isolate to all the seven antifungal tested while *Candida krusei, Candida tropicalis* and *Candida glabrata* were the most resistant species to all the seven antifungal tested. There was a statistically significant relationship between CD4 cell counts, oral hygiene, antibiotic usage, pregnancy and the prevalence of oral candidiasis in HIV/AIDS patients in this study.

### What is known about this topic

- The is increase prevalence of oral candidiasis in HIV/AIDS;
- Low CD4 count has been seen as a major risk factors for oral candidiasis.

### What this study adds

- Shows that the is increase resistance of *Candida* species to antifungals;
- Shows that poor oral hygiene and HAART without protease inhibitors remain a major risk factors for oral candidiasis in HIV/AIDS patients;
- Prevalence of Candidiasis in the South West Region, Cameroon.

## Competing interests

The authors declare no competing interests.
